# White Lies in Hand: Are Other-Oriented Lies Modified by Hand Gestures? Possibly Not

**DOI:** 10.3389/fpsyg.2017.00814

**Published:** 2017-06-22

**Authors:** Katarzyna Cantarero, Michal Parzuchowski, Karolina Dukala

**Affiliations:** ^1^Faculty in Sopot, SWPS University of Social Sciences and HumanitiesSopot, Poland; ^2^Department of Philosophy, Institute of Psychology, Jagiellonian UniversityCracow, Poland

**Keywords:** other-oriented dishonesty, white lies, body gestures, hand-over-heart, pre-registration, replication

## Abstract

Previous studies have shown that the hand-over-heart gesture is related to being more honest as opposed to using self-centered dishonesty. We assumed that the hand-over-heart gesture would also relate to other-oriented dishonesty, though the latter differs highly from self-centered lying. In Study 1 (*N* = 79), we showed that performing a hand-over-heart gesture diminished the tendency to use other-oriented white lies and that the fingers crossed behind one’s back gesture was not related to higher dishonesty. We then pre-registered and conducted Study 2 (*N* = 88), which was designed following higher methodological standards than Study 1. Contrary, to the findings of Study 1, we found that using the hand-over-heart gesture did not result in refraining from using other-oriented white lies. We discuss the findings of this failed replication indicating the importance of strict methodological guidelines in conducting research and also reflect on relatively small effect sizes related to some findings in embodied cognition.

## Introduction

*Chandler Bing*: Janice said “Hi, do I look fat today?” So I looked at her…

*Ross Geller*: Whoa, whoa. You looked at her? You never look. You just answer, it’s like a reflex. Do I look fat? No! Is she prettier than I am? No!

Friends (TV Series, 1994-2004)

We started writing this article with the idea to focus on the possible effects of the hand-over-heart gesture on refraining from using other-oriented white lies. The experiments presented in this article were in fact designed to test the idea that body gestures commonly associated with (dis)honesty influence white lies. We have drawn hypothesis from previous psychological research and then designed and conducted a study (Study 1) to put this hypothesis to the test. The study, however, was designed and conducted few years ago, following our best intention and using the knowledge we had back then, which, looking at it today, was not flawless. Due to recent crisis regarding replications of studies (see e.g., Open Science Collaboration, 2012, 2015; Klein et al., 2014; Hagger and Chatzisarantis, 2016) and especially with reference to the uncertainty regarding some embodied cognition effects (e.g., Ranehill et al., 2015; Wagenmakers et al., 2016), we felt that we should also make an effort to replicate our own findings. As we truly hope not to be chasing noise with our scientific endeavor, we have decided to pre-register the design and conduct a replication of Study 1. The replication held to higher methodological standards as compared to the original study. Thus this article shows first why we have developed the hypothesis we had and then, after presenting the research we have conducted and the results we found, we focus on both theoretical and methodological issues related to the (possible lack of) hand-over-heart gesture effect on other-oriented white lies.

## Embodied Cognition

The pure experience of reading a good novel is often thought to involve the feeling of being totally immersed in the whole multi-perceptual reality (by the means of visual imagery). Similarly, mere thinking about a concept is argued to involve simulating the relevant perceptual states (Barsalou, 2008; Bergen, 2015). According to the embodied perspective, people represent these concepts by the means of the same sensations that co-occur with the activation of such concepts (see Riskind, 1984; Chandler and Schwarz, 2009). Our bodies and their modalities can be perceived as grounding mechanism in cognitive processes (see Barsalou, 2009).

From very early on in our development, we make sense of social situations by assimilating their meaning with the states of our bodies, their movement or their orientation in space in those specific conditions. After many exposures, we learn how to associate the fact that giving somebody a hug means that you like that person or that pushing something away means that you do not like it. Similarly, a little child jumps around rapidly when faced with exciting stimulation. This kind of situated conceptualisation represents the configurations of multimodal components, that is, e.g., visual, auditory, proprioceptive, or interoceptive information that can be viewed as a specific perceptual pattern (Barsalou, 2009). The embodied cognition perspective suggests that when a component of a specific pattern is evoked or triggered, the remaining components are likely to be activated as well, as they form a pattern in which in the past they have frequently co-occurred with the perceived component. Hence, after many incidents where such modal patterns co-occur in quick succession, they are thought to form a unified situated conceptualisation in our memory that plays an important role in social cognition later in life (Barsalou, 2009). For example, the numeracy cognition observed among pre-schoolers is often based on their operations with fingers representing numbers in space (e.g., SNARC hypothesis – spatial-numerical association of response codes, Dehaene et al., 1993; Riello and Rusconi, 2011). After much exposure to such co-occurrence early in life, we can observe a pattern of spatial preference among adults as well – people respond faster to large numbers with their right answer key than with the left one, while small numbers are categorized faster with the left than with the right key (Dehaene et al., 1993).

The grounding theory (Barsalou, 1999, 2009; Niedenthal et al., 2005) proposes that simply increasing the accessibility of the specific concept (say the physical stimuli appears on left side of the visual field) can elicit thoughts, feelings, and judgments related to the concept that is applicable in this pattern (it would be appraised, judged, and coded as being smaller or of lesser value: Dehaene et al., 1993; Parzuchowski et al., 2016). For example, when hugging somebody – apart from signaling a liking for that person – it also involves a whole perceptual pattern of various sensations (experience of warmth, the smell of the person, softness of their skin, and so on). Thus when people experience warmth (e.g., when they are placed in a warm room), they are more likely to perceive others as friendly and kind (IJzerman and Semin, 2009, 2010; Szymkow and Parzuchowski, 2013; Szymkow et al., 2013; IJzerman et al., 2016).

It is important to note that these bodily induced association activations are thought to take place unobtrusively, and even without awareness of their semantic meaning (see Chandler and Schwarz, 2009; Jostmann et al., 2009). For instance, Chandler and Schwarz (2009) claimed to study text comprehension while instructing participants to perform various finger movements. During this experiment they asked participants to extend their middle fingers (a hostile gesture), or to extend their index fingers (a neutral, control gesture). The participants were asked to indicate their impressions of an ambiguously described person while performing the finger movement (hostile or control gesture). None of the participants noticed that they had, in fact, been performing the valenced gesture. Interestingly, those making the hostility–associated gesture perceived the target person as more hostile than the controls did.

To sum up, many research findings appear to show that merely experiencing a bodily sensation associated with certain concepts is enough to shape subsequent information processing, although the pattern of associations related to the body manipulation should be limited to the previous repetition pattern and its recognition. Thus the effects of such bodily manipulation are at the same time sensitive to culture and contextual clues (for a discussion on this topic, see Bialobrzeska and Parzuchowski, 2016). There is therefore theoretical and empirical evidence in psychological literature that indicates the effects of embodied cognition. We next turned to the literature on dishonesty and its’ link with embodied cognition, as we wanted to focus on gestures related to dishonesty.

## Self-Oriented And Other-Oriented Dishonesty

People in long-term relationships would often agree with the anecdotal advice coming from a fictitious character, Ross Geller and mentioned at the beginning of this article. When sensitive questions are being asked by the partner (e.g., “*Do I look nice in that dress/suit*”), one should not take time to give an informed response (“*Well, let me see*”). Many would agree that the highest scoring response will be a prompt and firm confirmation (“*Yes, you look great in everything*”) to prevent any type of unwanted discussions that a hesitation may trigger. We often lie for the sake of our relationships with others. Yet even though people report having lied at least once a day, most of our daily communication is free from deception (DePaulo et al., 1996).

Interestingly, contextual cues may trigger the tendency to give more honest responses. Previous research shows that emblematic gesture manipulation (namely hand-over-heart) can induce a more honest response regarding the way we behave or judge ourselves and others (Parzuchowski and Wojciszke, 2014; Parzuchowski et al., 2014, 2017). However, the hand-over-heart gesture has been proven to elicit a more honest approach mainly in perceiving other’s intentions (Parzuchowski et al., 2014, 2017) or *self-oriented* motivational contexts (Parzuchowski and Wojciszke, 2014). Namely, people were less inclined to be dishonest to benefit themselves when they posed the hand-over-heart gesture.

In the present article, we were interested in verifying the application of this gesture when dishonesty is motivated more *prosocially*. There are many ways of differentiating the types of lies (see, e.g., Camden et al., 1984; Arcimowicz et al., 2015). One of the most important categorization depends on the type of the beneficiary of the lie (e.g., DePaulo et al., 1996; Erat and Gneezy, 2011). The primary beneficiary of the lie can be the liar, another person or both. There is strong evidence showing that in fact self-oriented (or self-centered) and other-oriented dishonesty are significantly different from each other (e.g., Kashy and DePaulo, 1996; Cantarero and van Tilburg, 2014).

While self-oriented lies are the ones that are primarily aimed to benefit the liar (DePaulo et al., 2004), other-oriented lies aim at providing benefit to another person (DePaulo et al., 1996). White lies (or Pareto white lies) are aimed to benefit both the liar and another person (Erat and Gneezy, 2011). They are related to the willingness to be polite and to care about another person’s feelings. The benefits of another person related to these lies can involve trying to make another person feel good by saving them from an unpleasant truth. On the other hand, white lies also bring benefits to the liar, like maintaining good interactions with others, being perceived as a nice, good person, or being liked. Nevertheless, in white lies, the other-oriented motivation is more important than in self-oriented dishonesty one. In the present article, we wanted to focus on lies that are not primarily aimed to benefit the liar, that is, on other-oriented white lies that include the interests of others.

The decision of whether to lie or to tell the truth depends on the consequences that a discovered lie has (e.g., Mazar and Ariely, 2006). Other-oriented lies, when unraveled, can be argued to have less severe consequences (Arcimowicz et al., 2015). Lies that are aimed at bringing benefits to others are also far more acceptable than the ones that are centered on the benefits of the liar (Lindskold and Walters, 1983). As a result, the cost-benefit analysis of the decision as to whether to lie or to tell the truth should differ depending on whether a lie is self-centered or there is other-oriented motivation involved. Since the psychological costs of lying are much lower for other-oriented lies, we should expect the decision of whether to use such lie to be much easier, than when a lie is self-centered. Interestingly though, when we lie, most of our lies are self-centered and not other-oriented (DePaulo et al., 1996). It seems that we are willing to accept more psychological costs of lying in exchange for receiving more personal benefits that a lie might bring.

This poses an interesting dilemma related to truth telling and lying. Previous studies have indicated that self-oriented and other-oriented dishonesty might relate in an adverse way to a self-regulation process (Cantarero and van Tilburg, 2014). Namely, while ego-depletion promotes self-centered dishonesty (Mead et al., 2009), the same conditions should push us toward reduced proneness to other-oriented deception (Cantarero and van Tilburg, 2014). This is due to the assumption that other-oriented dishonesty demands more effort and is less of a ‘default’ option for people. Should using an unobtrusive hand gesture be related to other-oriented dishonesty? As previously mentioned, the hand-over-heart gesture when performed by a target person he/she appeared more trustworthy than the same targets photographed with both hands down. Using the hand-over-heart gesture lead to refraining from self-centered dishonesty (e.g., Parzuchowski et al., 2014). Presumably, when the gesture is incorporated by the agent (unobtrusively within some other bogus task), it serves as the implicit association with the honesty and trigger participants to behave accordingly. Just as Mazar et al. (2008) showed that swearing an oath of allegiance to a bogus honor code or attempting to recall norms (The 10 Commandments), made people act in a more honest way, presumably because this drew attention to one’s internal standards of integrity. One could argue that since polite, white lies are so socially acceptable, they pose almost no dilemma in the communicator and thus people will not refrain from telling such lies even when placing their hand over their heart.

In study 3, Parzuchowski and Wojciszke (2014) have shown that placing a hand over the heart caused people to withhold their honest opinion about the (un-) attractiveness of the alleged acquaintances of the experimenter. Participants were asked to rate how attractive people presented in the photos were. This information was not given in the presence of the judged person or in a face-to-face setting. As a consequence, such a situation might not have engaged much of the interest of the interlocutor. In the presented study, we address these issues and add new insights into the role of using hand gestures in promoting and refraining from dishonesty. In the study presented below, we wanted to test whether a white lie aimed at protecting others from harm will depend on a performing a gesture related to honesty, namely the hand-over-heart gesture.

In order to reach this goal, the design of the presented experiments involved the presence of the supposed author of considered artworks. We focused on the disliked artworks of the ‘author’ and asked participants to give their feedback about the work to the face of the alleged author, similarly as in the study by Bell and DePaulo (1996). We introduced this setup to trigger a dilemma for our participants between telling the blunt truth about their aesthetic dislike, or acting politely and expressing the alteration of their ratings. This is a clear situation that should involve other-oriented white lies – social norms should trigger expression of less extreme preferences when faced with the author in order to spare him/her from feeling bad. This motivation to lie is other-oriented especially because the participants did not expect to interact with the alleged author after the experiment and telling the truth could possibly hurt the feelings of the author.

Our goal was to test if gestures related to (dis)honesty can influence one’s particular social behavior – tendency to tell other-oriented white lies aimed at protecting others from harm (namely to exaggerate ones’ aesthetic judgments about artworks). Additionally, in Study 1, we wanted to see whether it is gestures that promote and prevent lying that will affect telling white lies. For this reason, in Study 1 we also included the fingers crossed behind one’s back gesture that should augment lying behavior. Previous studies have shown that the hand-over-heart gesture reduces self-centered dishonesty (e.g., Parzuchowski and Wojciszke, 2014). We wanted to test whether the use of the gesture primes other-oriented honesty by measuring whether people are more likely to give true (non-flattering) feedback to others (unfamiliar author of a work of art). More specifically, we wanted to focus on other-oriented white lies and hypothesized that the tendency to use these lies will be diminished when performing the hand-over-heart gesture. The second aim was to check if the opposite gesture (fingers crossed behind one’s back) promotes dishonesty, we hypothesized that it would prime people to give more positive but untrue feedback to others. Additionally, we wanted to control the degree to which the ‘author’ was liked, as it should be related to higher proneness to use other-oriented lying, which was the case in former studies (e.g., Bell and DePaulo, 1996). To test the hypothesis we conducted a laboratory experiment (Study 1) and then conducted a replication study (Study 2) that was focused solely on the hypothesis regarding the hand-over-heart gesture and the other-oriented white lies.

## Method

The main aim of the studies was to test whether the hand-over-heart gesture is related to refraining from using other-oriented white lies.

## Study 1

### Participants and Design

Eighty-three university students (67 women; *M*_age_ = 20.92, *SD* = 1.61) participated in the experiment in exchange for course credit. Participants in the study were enrolled to participate via a campaign advertising the study as ‘*Body posture and perception of artistic work.’* The study was run in individual sessions, and each lasted around 30 min. All participants gave their informed consent. At the end of the procedure, participants were asked to guess what the purpose of the research was. Data from the four participants that guessed the correct hypothesis were excluded from the analysis and thus the total sample consisted of 79 people.

The procedure of the experiment was adapted from Bell and DePaulo (1996). The laboratory was turned into an ‘art gallery’ displaying 10 photos (which were numbered from 1 to 10). Participants were randomly assigned to one of three experimental conditions (hand-over-heart, fingers crossed behind the back, or a control gesture: hand over elbow, see Appendix 1). In each of the experimental conditions, participants were asked to pose the main gesture and two other gestures (these were: hand over arm, hand over hip, see Appendix 1) while evaluating the artwork (the order of the gesture use was counterbalanced). We refrained from using the expression ‘hand-over-heart,’ to avoid having the possibility of receiving the effect by means of simply instructing participants to behave according to the meaning of the gesture. In the hand-over-heart condition participants were asked to place the hand at a given height of their chest. We did this to distract the participants from the factual aim of the study. Participants were instructed to pose these gestures after having heard a verbal signal that described it. To standardize the instruction, the verbal signals of the gestures were pre-recorded and were played to participants during the experiment.^1^

There were always two experimenters conducting the study. Experimenter 1 was mostly responsible for conducting the first part of the story and Experimenter 2 was conducting the conversation about the photos after being introduced as an alleged author of some of the artworks. Firstly, in order to present the cover story, participants were asked to, first, privately assess without verbal or written statement one by one all of the photographs presented in the ‘gallery,’ while rotating gestures in accordance with the pre-recorded verbal signals played from the speakers. Only then were participants given a piece of paper and asked to choose two photos from the gallery: the one they liked the most and one they liked the least and to evaluate both chosen pictures on a Likert scale from 1 to 7, where 1 was “*Definitely don’t like it*” and 7 was “*Definitely like it.*” After having the participant evaluate the photos on paper, Experimenter 1 would pass that information to Experimenter 2 (without being noticed by the participants), so that s/he knew which photos to talk about with the participant. The participant was then asked to back up his or her opinion (in writing) stating why he or she had chosen the ‘favorite’ and the ‘worst’ photographs (while writing the evaluations participants were not posing any gestures).

At this phase of the experiment, seconds after having written their evaluations of the photos, participants were asked to talk to the alleged author (the Experimenter 2) of some of the photos that were presented in the ‘gallery.’ Experimenter 2 was blind to the hypothesis of our study. We said that, in the experiment, we were also interested to know how people discuss artwork verbally while posing gestures. We also told participants that this would be useful feedback for the “author.” At this point, the auditory instruction (‘chest,’ ‘fingers,’ and ‘elbow’) was played and we asked the participant to hold the gesture during the whole conversation with “the author” (Experimenter 2). At this stage Experimenter 2 (alleged author) entered the room and asked participants about three photos. The questions were the same every time (whether the participant liked a photo on a 1–7 scale, where 1 was “*Definitely don’t like it*” and 7 was “*Definitely like it*”; then two open-ended questions were asked: to justify their evaluation and to describe the topic of the photo). The first two photos to be evaluated were random ones and not that were not indicated by the participant as the most liked one or the most disliked one in writing a few minutes earlier. The third photo was always the one that the participant chose as the most disliked one in the phase of the written evaluations of the photos. The evaluation of this third photo in conversation, the one we knew was the disliked one (and evaluated in writing a couple of minutes earlier), was the main interest of the experiment and our main operationalisation of the dependent variable.

Afterward, we asked the participant to evaluate both of the experimenters (we said that we would appreciate the feedback from each participant regarding the experiment). The questions that we used were for example: ‘*I think that this person was professional’* (this was the mock phrase, used to make the assessment of the experimenters seem more credible to participants), ‘*I think that this person is nice.’* The answers were on a 1 (‘*Definitely yes*’)–7 scale (‘*Definitely not*’). The questions came from Kulesza et al.’s (2015) liking scale.

We then thanked the participants for their participation, gave the credit points and after we had examined all the participants, we debriefed them. We expected that higher ratings given in the oral evaluation phase of the experiment (compared to the first, written evaluations) would indicate that the situation indeed promoted lying (i.e., giving an excessively positive feedback on artwork to an alleged author). We assumed that participants would be less eager to lie when placing their hand over their heart. We assumed that the participants would be more willing to lie in the oral evaluation while presenting the fingers crossed gesture.

### Results

**FIGURE 1:**
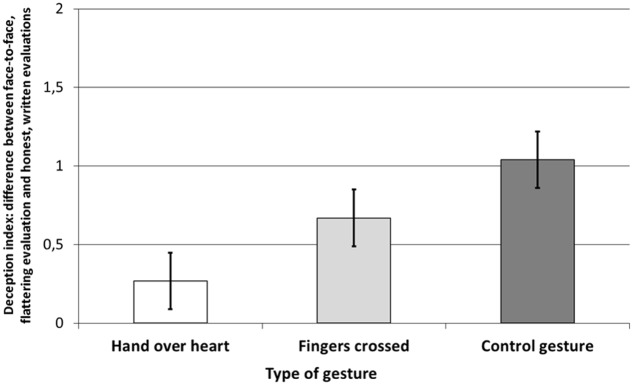
**Aesthetic judgments of the ‘worst photo’ as a factor of the type of gesture (control gesture, *N* = 26; fingers crossed, *N* = 27; hand-over-heart, *N* = 26).** The results show the deception index – the difference between the oral, face-to-face, more flattering evaluation and the written and honest evaluations of the same, least liked photo, as communicated to the alleged author of the photo. Error bars present standard errors.

The six items liking scale (after exclusion of one mock item) reached a good reliability coefficient of *α* = 0.76. We then calculated an index of deception, which was the difference between the second, oral evaluation of the least liked photo and the first, written evaluation of the same, least liked photo.^2^ A one-way ANCOVA was conducted with the deception index as a dependent variable, liking of the alleged author as a covariate and type of gesture (hand-over-heart, fingers crossed, and hand over elbow) as a between-subjects factor. The results showed a main effect of the gesture *F*(2,75) = 5.16, *p* = 0.008, η^2^ = 0.12.^3^ Comparison of means with Bonferroni correction showed that there was a significant difference between the hand over elbow gesture (*M* = 1.04, *SD* = 0.92) and hand-over-heart (*M* = 0.27, *SD* = 0.78), *p* = 0.006, while the fingers crossed gesture results did not differ from the aforementioned significantly (*M* = 0.67, *SD* = 1.07). Lower deception index in the hand-over-heart condition means that participants were indeed less likely to use an other-oriented lie when performing the gesture, which supports our hypothesis. The fingers crossed behind one’s back, however, did not promote lying, leaving our second hypothesis without support.^4^ These results are presented in **Figure 1**.

**Table 1:** Descriptive statistics of the evaluations of the two most disliked photos and two photos of more attractiveness of +1 SD.

Type of characteristic	Disliked photo A		Disliked photo B		Liked photo A		Liked photo B	
				
	*M*	*SD*	*M*	*SD*	*M*	*SD*	*M*	*SD*
Liking	2.54	1.34	2.66	1.48	4.62	1.58	4.48	1.53
Quality	3.82	1.51	3.78	1.50	4.92	1.44	4.95	1.29
Professional photo	2.90	1.34	2.92	1.33	4.09	1.60	4.07	1.47
Author professional	2.75	1.27	2.86	1.34	3.93	1.63	3.89	1.45


The results also revealed that there was a trend regarding the likeability of the author *F*(1,75) = 2.87, *p* = 0.094, η^2^ = 0.04. Further analysis showed a trend that indicated that the more the alleged author was liked, the higher the deception index was *r*_s_(79) = 0.16, *p* = 0.077 (one-tailed).^5^ It suggests that people may be more likely to give positive, even if not true, feedback to those whom they like more. The liking of the alleged author did not differ between the experimental conditions (*p* = 0.183).

The results of Study 1 suggested that the hand-over-heart gesture is related to refraining from using white lies comparing to control gesture. We wanted to replicate these findings and conducted a study that was of similar design to Study 1. We focused solely on the hand-over-heart gesture in Study 2 and decided to improve the design of the study. We intended to make both the procedure and the study material better. With the new design, we have, among others, excluded the part, where Experimenter 1 unnoticed passes information to Experimenter 2. The following studies were pre-registered at osf.io.^6^

## Study 2

We preceded Study 2 with an additional pilot study to choose the appropriate stimuli.^7^ The aim of Study 2 was to replicate the main finding from Study 1.

### Participants

Eighty-eight participants (65 women, *M*_age_ = 29.27, *SD*_age_ = 10.21) took part in this study in exchange for course credits.

### Materials and Procedure

We invited our participants to join a two-part study. The first study one was said to be aimed at investigating the way people talk about photographs. We said that the second study would be on further evaluation of research material. Upon the arrival to the lab, participants were asked by Experimenter A (blind to the hypothesis of the study) to take part in a supposedly unrelated, third study (with additional small credit given to all participants willing to join the short study) where the new breathing measurement device needs to be calibrated for an upcoming student project for sport psychology. The new breathing measurement device supposedly involved having a chest rubber band that needed to be tightened with the use of the shoulder (resulting in a hand over shoulder gesture) or with the right hand (resulting in a hand over heart gesture). This information allowed us to have participants perform the gestures without being aware of the factual aim of the study. Participants were then asked to simply have the device placed on their body for 20 s and then Experimenter A started recording their breathing rhythm during the remaining time spent in the lab (in order to calibrate the measurements of the device). After that Experimenter A left the participant with Experimenter B.

Experimenter B (blind to the hypothesis of the study) then explained that she was a fellow student but in her free time she took photo class and that for her Master thesis qualification she was pursuing some qualitative research interested in how people talk about photographs. She explained that for the purpose of the study she will ask participants to rate pictures taken by her and other students that pursue the hobby of taking pictures as a part time class.

The photos were then displayed at a computer screen one, by one. The participants always first saw the two mildly unattractive photos (in random order) and then the two most unattractive photos (in random order). The pictures appeared with their authors and numbers below them, to make it easy to refer to them. Experimenter B asked separately about each of the photos (saying its’ number and author) whether the participants liked it on a 1–7 scale, where 1 stood for *definitely not* and 7 stood for *definitely yes*. She then asked what the participant liked about the photo and what s/he disliked about it. Each time a third photo came up, the Experimenter B would inform the participant that in fact she was the author of the presented photo.

Participants were then asked to stop the breathing measurement (and release the gesture) and answer via computer open-ended questions regarding how they had felt when talking about art, whether they had used the type of words they typically use when they discussed the photos and whether they had felt comfortable talking about the photos or anything unusual happened during the procedure. These questions were used to probe for hypothesis guessing and cover up the real aim of the study. Participants were then asked demographic data.

After 1 week from the main part of the study, participants were asked to fill an online second part of the study. Participants were presented with 10 photos consisting of the four pictures from the main part of the study and other six that came from the pilot study. Among the six pictures, three were evaluated in the pilot study as the most liked ones, two were of average liking and there was also one of low evaluations (as shown in the pilot study). Participants were asked to rate on a 1 = d*efinitely not* to 7 = *definitely yes* scale whether they liked the photo, considered it of good quality, perceived it as a photo professionally taken, thought that it was taken by a professional and whether they had ever seen this photo before. We then gathered demographic data and asked about the perceived aim and hypothesis of the study. After the study participants were fully debriefed.

### Results

We conducted repeated measures ANOVA with liking of the photo as a dependent variable, type of evaluation (face to face vs. online afterward) and authorship of the photo (unknown vs. the interlocutor) as a within-subject and type of gesture (hand-over-heart vs. hand over arm) as a between-subject factor.

We found the main effect of the type of evaluation, *F*(1,73) = 44.69, *p* < 0.001, η^2^ = 0.38. Participants gave more favorable evaluations in person (*M* = 4.11, *SD* = 1.41), than in an online setting (*M* = 3.35, *SD* = 1.54). There was also the main effect of the authorship *F*(1,73) = 5.88, *p* = 0.018, η^2^ = 0.08. When participants were informed that they were talking to the ‘author’ of a photo, they declared higher liking of that photo (*M* = 3.89, *SD* = 1.49), than when they were not talking to the alleged author (*M* = 3.57, *SD* = 1.51). There was no significant main effect of the gesture *F*(1,73) = 0.02, *p* = 0.900. There was no significant interaction between the type of gesture and the authorship *F*(1,73) = 0.08, *p* = 0.781.

We only found a significant interaction between the gesture and the type of evaluation *F*(1,73) = 10.15, *p* = 0.002, η^2^ = 0.12. Comparison of means with Bonferroni correction revealed that in the hand-over-heart condition participants gave more positive evaluations in person (*M* = 4.30, *SD* = 1.31) to the online evaluations (*M* = 3.20, *SD* = 1.44), *p* < 0.001. Similarly so, in the hand over arm condition evaluations in person were higher (*M* = 3.91, *SD* = 1.50) than the online ratings of liking (*M* = 3.51, *SD* = 1.64), *p* = 0.016.

**FIGURE 2:**
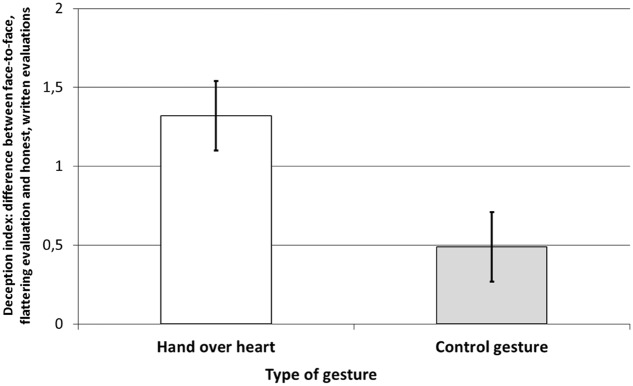
**Aesthetic judgments of the photo ‘authored’ by the experimenter as a factor of the type of gesture (control gesture, *N* = 37; hand-over-heart, *N* = 38).** The results show the deception index – the difference between the oral, face-to-face, more flattering evaluation and the written and honest evaluations of the same photo. Error bars present standard errors.

We conducted an additional analysis, similar to that used in Study 1. We first calculated a deception index, which was the difference between the face-to-face evaluation and the ratings made online. We then conducted *t*-test analysis with the experimental condition as an independent variable and the deception index as a dependent variable. Results showed a significant main effect of the experimental manipulation *t*(73) = 2.64, *p* = 0.010, *d* = 0.61. Surprisingly, the deception index was higher in the hand-over-heart condition (*M* = 1.32, *SD* = 1.38), than the hand over arm (*M* = 0.49, *SD* = 1.35). These results are graphically displayed in **Figure 2**.

## General Discussion

We hypothesized that participants who used the hand-over-heart gesture would be more likely to give true feedback to “authors” of the work of art. The results of Study 1 showed that participants were more honest (less flattering) while performing the hand-over-heart gesture. However, the results of Study 2 showed no difference between the hand-over-heart gesture and the control gesture in the proneness to use other-oriented white lies regarding a photo that was either authored by the interlocutor or of an unknown authorship. What is more, when we conducted a similar analysis as in Study 1, we found the deception index to be significantly higher in the hand-over-heart condition to the control group. These inconsistent results raise several important issues of both methodological and theoretical kind.

### Methodological Concerns

We designed and conducted Study 1 a few years ago, using our best knowledge and intentions. We do recognize, however, that the study had imperfections and we had not pre-registered our experimental protocol. The design was unnecessarily complex, we did not calculate the sample size before conducting the study and relied on the rule of thumb instead. We also could not exclude the possibility that there could be an influence of posing gestures in the first stage of the experiment on evaluations and preferences regarding the photos. A *post hoc* analysis of the preferences did not show any patterns in preferences depending on the condition, limiting such a possibility, yet without excluding it. What is more, it would have been better if Experimenter B had not known the preferences of each of the participants while talking to each of them.

We thus designed and conducted Study 2. We wanted to focus solely on the hand-over-heart effect on other-oriented white lies hypothesis and replicate previous findings, yet this time using a better design. Of course Study 2 is not free from limitations. Most importantly, we failed to gather the desired number of participants. We aimed at reaching 114 participants, when we could only gather 88 (in the time limit we had), out of which 75 took part in the follow-up assessment of the photos. This is clearly a drawback of the study. However, we also conducted a similar analysis to that in Study 1. Namely, we decided to conduct an additional analysis relying on the deception index. *Post hoc* analysis regarding the attained power showed that the achieved power was 0.83. This implies that the number of participants we gathered should be enough to detect the effect, had we wanted to rely solely on the deception index.

Furthermore, there were other differences between Studies 1 and 2 that might have affected the results, although based on taxonomy developed by Hendrick (1991) the methodological similarities between Studies 1 and 2 would have to call it a fair replication. We used a different cover story to introduce the necessity to pose the hand-over-heart gesture. We do think, however, that should the lack of effect of the gesture be the result of the different cover story applied, it only points to the fact that the effect (should it exist), is much weaker than we originally thought. Importantly, in Study 2 we gathered replies on personal evaluation of the photos 1 week *after* having stated verbally in front of the alleged author the extent, to which one liked the photo. It is possible that when participants are in the situation of just having judged a photo as unattractive (which they did in Study 1), they are more aware of the conflict between social norms and the norm of being honest and thus such a setting might have a stronger influence. Yet, both experimental settings did promote giving over positive feedback to the alleged author, which suggests that the argument of the moment in time when we gather private opinions about the photo should not affect much the hypothesized effect.

### Theoretical Implications

The hypothesis presented in this paper were driven from psychological theory and research findings supporting it. We think that it would be most correct to draw careful conclusions regarding theoretical implications. It is important to note, that we conducted only one replication of that effect. A series of multilab studies would give more solid grounds to be able to talk about robustness of an effect. We do think that above all, the results of the studies presented in this article point to the fact, that should the hand-over-heart gesture indeed influence the tendency to refrain from other-oriented white lies, it would probably be a much weaker effect. Changes in the setting between Studies 2 and 1 might have altered the obtained results, yet as stated previously, this would only imply the weakness of the original effect.

We need to point to an important theoretical issue regarding the subject of the study. We knew that the hand-over-heart gesture is related to refraining from *self-oriented* dishonesty (e.g., Parzuchowski et al., 2014). We therefore wanted to verify whether the effect could be generalized for other-oriented white lies. These kinds of lies are prosocially oriented and their aim is to protect others and to make the other person feel good, or at least spare them from feeling bad. People often find such lies to be even more ethical than truth telling (Levine and Schweitzer, 2014). Also, telling such a polite lie is behaving in a socially acceptable way. A rough truth is socially less acceptable than a prosocial lie (Levine and Schweitzer, 2014) and social influence mechanisms indicate that being liked is a powerful tool in social interactions. It is possible that because other-oriented white lies are so socially acceptable, they are thought of as less of a lie and therefore they do not trigger such a strong dilemma as is the case with self-oriented lying. Namely, it might be that participants do not feel that there is a problem with giving an over positive feedback, as it is not so much lying when it serves another person. We thus limit the generalization of our findings to other-oriented white lies and indicate that the type of lie that was under study might be a significant factor that influenced the results.

We did find an unexpected result that indicated higher deception index in the hand-over-heart to the control group. We do not find any theoretical support for this result. If anything, such result points to the fact that the effect of hand-over-heart on other-oriented white lies either does not exist, or is extremely weak and sensitive to delicate contextual changes.

## General Conclusion

In recent times several social priming/embodied effects came under scrutiny, e.g., cleanliness priming (Johnson et al., 2014), elderly priming (Doyen et al., 2012) or power posing (Garrison et al., 2016; online databases tracking their recent replications are PsychFileDrawer.org and CurateScience.org). Overall, we do not concur with the position that the effects of embodied cognition are in general doubtful. We are convinced that there is a significant body of compelling and replicable evidence (e.g., switching cost paradigm, Pecher et al., 2003) for the inclusion of sensorimotor system in cognitive processes (see e.g., Wilson, 2002; Pecher and Zwaan, 2005; Fischer and Zwaan, 2008; Glenberg, 2010). It has also been argued that replications of existing effects sometimes produce non-significant results (Cohen, 1969) and that (mis)replications are sensitive to contextual factors (see Van Bavela et al., 2016). That said, we are doubtful of the effect tested within this article – the tendency to use other-oriented lies is possibly not affected by honesty activation. To conclude, at least when effects are small, high methodological standards (e.g., high power, blindness to hypotheses and probing for hypothesis guessing) are vital in distinguishing when one is in search of an interesting hypothesis and when one is chasing noise. Though we cannot be entirely sure, it is possible that in our case it was the latter.

## Ethics Statement

The study was approved the Ethics Committee (decision number 1/03/2012) at the SWPS University of Social Sciences and Humanities, II Department of Psychology in Wroclaw. In all of the studies reported here, participants were informed that their participation was totally voluntary and that they could resign from the study at any step of it. They were also informed that the data that was gathered in the study was confidential and used for scientific purpose only.

## Author Contributions

Study 1: KC designed the work, KD acquired the data KC did the analysis, KC, KD, and MP interpreted the data for the work. KC, KD, and MP drafted the work and revised it critically for important intellectual content. KC, KD, and MP approved of the version to be published. KC, KD, and MP agreed to be accountable for all aspects of the work in ensuring that questions related to the accuracy or integrity of any part of the work are appropriately investigated and resolved. Study 2: The pilot study was prepared by KC, KD and MP, run by KC and main analysis were conducted by KC. The main study was prepared by MP, KD, and KC, run by MP and his research assistants and main analysis was conducted by KC. The write-up of the results was conducted by KC. Each contributor participated in preparation of the final form of the research report and final paper describing the results.

## Conflict of Interest Statement

The authors declare that the research was conducted in the absence of any commercial or financial relationships that could be construed as a potential conflict of interest.

## Footnotes

1 The verbal signal was changed every 15 s. The order of the verbal signals presented to the participants was identical across conditions with the exception of the key gestures that were under study (hand-over-heart vs. fingers crossed behind one’s back, vs. hand over elbow): hip, arm, (key gesture), arm, (key gesture), hip, arm, hip, (key gesture), arm. That is, the first verbal signal was ‘hip,’ when the participant heard the word they were supposed to place their hand over their hip while evaluating a photo. Then they were asked to turn to another photo when they heard a new verbal signal (and change their gesture). If the participant was in the ‘hand-over-heart’ condition, they would hear the word ‘chest’ as the third signal. In such an instance, a participant would turn to the next photo and place their hand over their heart. The examples of the gestures are presented in Appendix 1.

2 For the sake of clarity and brevity in the main body text, we decided to present the findings using a deception index. However, a repeated measure mixed-model ANOVA, with oral and written evaluations as within-subjects factors and type of gesture as between-subjects factor, yields practically the same results. The difference between oral (*M* = 2.38, *SD* = 1.15) and written (*M* = 1.72, *SD* = 0.90) evaluation is significant, indicating that the experimental setting did promote lying *F*(1,76) = 39.34, *p* < 0.001.

3 Results of the ANOVA analysis without the covariate yield similar results. Namely, the experimental manipulation does show a main effect on the deception index, *F*(2,76) = 4.26, *p* = 0.015, η^2^ = 0.10. Comparison of mean replies with Bonferroni correction indicates significant differences only between the control and the hand-over-heart condition (*p* = 0.012).

4 We ran a supplemental study afterward to test the recognisability of the gestures used in the main study. The reason behind this identification test is that the level of gesture recognition could be a proxy for the strength of the association between the bodily movement people perform while making the gesture and the concept that it is represented being activated in the same time. Eighty-four pedestrians in Cracow, Poland (48 women, *M*_age_ = 23.47; *SD*_age_ = 4.17) were asked one dichotomous question about a gesture’s familiarity (“*Are you familiar with the gesture?*”; answers were either “yes” or “no” while the experimenter presented the gesture), and one open-ended question about the knowledge of the context the gesture is used (“*Do you know in which situations this gesture would be used?*”). We also asked participants to rate how frequently they use the target gesture (“*How often do you use this gesture yourself?*”) on a scale from 1 (never) to 7 (always). Half of the participants were presented the accompanying hand-over-heart gesture and half of them the fingers crossed behind one’s back gesture. A χ^2^ test showed a significant difference in judgments of familiarity between the gestures used: χ^2^(1) = 6.89, *p* = 0.009, *v* = 0.29. When asked about the hand-over-heart gesture, more participants declared knowing it (42.9% of participants, 18 people claimed that they know the gesture,) than when asked about the fingers crossed gesture (16.7% of participants; 7 people). Next, we coded open-ended answers on the knowledge of the context the gesture was used. Participants’ responses were divided into two groups ‘1’: ‘recognized’ (i.e., answers “when lying” in the case of the fingers crossed gesture and “when telling the truth” or “to be perceived more truthful” in the case of the hand-over-heart gesture) and ‘2’: – ‘not recognized’ (or any other answers, such as “don’t know,” “for a laugh” or “doesn’t matter”). A χ^2^ test showed that more people correctly recognized the hand-over-heart gesture (76.2% of participants; 32 people) than the fingers crossed gesture (52.4% of participants; 22 people), χ^2^(1) = 5.19, *p* = 0.023, *V* = 0.25. Ratings of frequency of gesture usage were analyzed using the independent *t*-test, which showed a significant difference between declared frequency of using the hand-over-heart and fingers crossed gestures, *F*(1,82) = 12.99, *p* = 0.001, η^2^ = 0.14. Participants declared using the hand-over-heart more often than the fingers crossed gesture (*M* = 3.57, *SD* = 1.23 and *M* = 2.57, *SD* = 1.31, respectively). This suggests that the hand-over-heart gesture is better known than the fingers crossed behind one’s back gesture. Also, people seem to be more aware of the context in which the gesture is used. Finally, people declare that they are more likely to use the hand-over-heart gesture than the gesture of fingers crossed behind one’s back.

5 There was also a positive correlation between the liking of the alleged author and the extent to which the oral evaluation of the author’s photo was positive, *r*(79) = 0.24, *p* = 0.016.

6 https://osf.io/vkpdb/

7 We also conducted a pilot study to be able to choose the best research material for the Study 2. Two hundred and seventy four students (*M*_age_ = 24.69, *SD*_age_ = 7.49) took part in an online study on evaluation of research material. The sample consisted of 213 women and 59 men. We have prepared photographs of landscape. The photographs were aimed at being of rather medium to poor quality. After gathering informed consent, participants were asked to evaluate a set of photos. Thirty photos were then randomly displayed to participants. Participants were asked to answer to four questions regarding each of the photos. We asked about the extent to which participants liked the photo, considered it of good quality, perceived it as a photo professionally taken and finally we asked whether participants thought that the photo was taken by a professional. All of the questions were answered on a Likert-type 1 to 7 scale, where 1 = *definitely not*, and 7 = *definitely yes*. We analyzed mean replies of the thirty photos regarding the four questions. We focused on two pictures that would be most disliked and not differ regarding the evaluation of the three additional questions. We then also picked two photos that would be at least 1 SD liked more that the two most disliked ones. We also wanted the more liked photos to be evaluated more favorably than the disliked photos regarding the other qualities, while at the same time be of similar evaluation among each other. This allowed us to pick two photos that were equally negatively evaluated and two photos that were evaluated positively. **Table 1** presents descriptive statistics of the four photos. We have analyzed mean evaluations of the four chosen photos regarding the four characteristics. We then calculated a series of dependent *t*-tests. We found that the photos chosen as the disliked ones did not differ between each other regarding liking, quality, perceived professionalism and perceptions of whether the photos were taken by a professional. At the same time the more liked photos did not significantly differ between each other regarding the four characteristics. We calculated two indexes of the evaluation: one comprised of the evaluations of the four characteristics of the two disliked photos (Cronbach’s α = 0.85) and one comprised of the evaluations of the two more liked photos (Cronbach’s α = 0.86). We then conducted a final dependent *t*-test analysis and found that the disliked photos in overall were more negatively evaluated (*M* = 3.03, *SD* = 0.97) than the more liked photos (*M* = 4.37, *SD* = 1.06), *t*(273) = 20.10, *p* < 0.001, *d* = 1.22. We used the four photos in Study 2.
