# Supplementary material for: White Lies in Hand: Are Other-Oriented Lies Modified by Hand Gestures? Possibly Not
Source: Front Psychol. 2017 Jun 22;8:814. doi: 10.3389/fpsyg.2017.00814 (PMC5479878; doi:10.3389/fpsyg.2017.00814)
Supplement: Supplementary file 1 [file Data_Sheet_1.docx]

Appendix 1

Examples of gestures performed during the study: (1) Hand-over-heart; (2) Fingers crossed behind one’s back; (3) Hand over elbow; (4) Hand over arm; (5) Hand over hip.

| 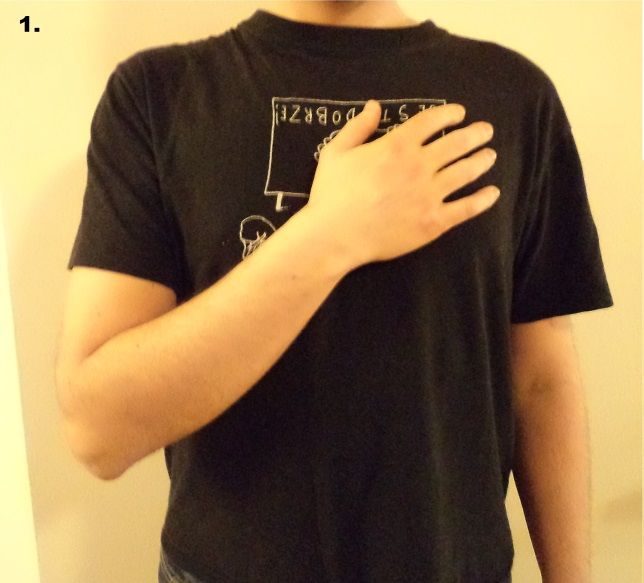 | 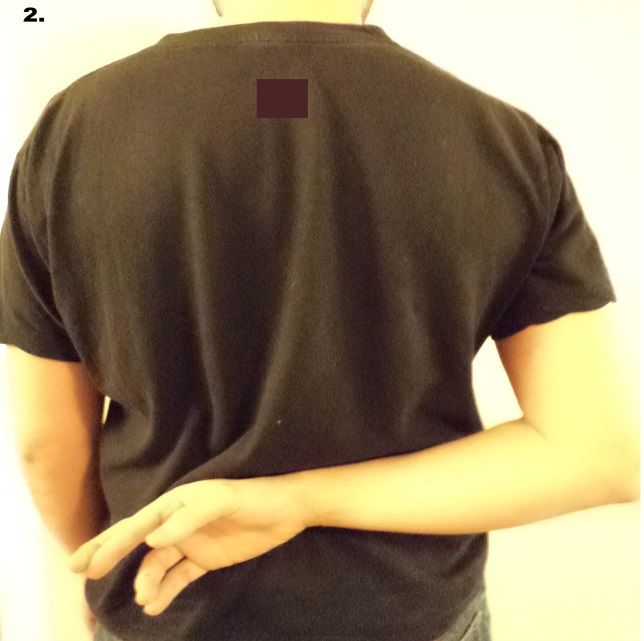 |
| --- | --- |
| 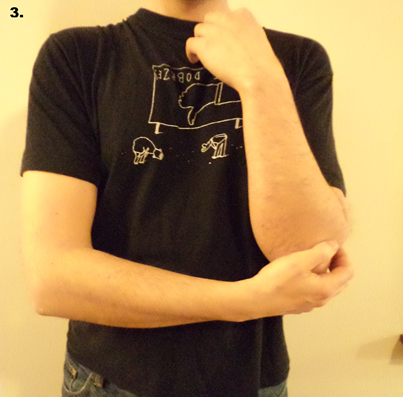 | 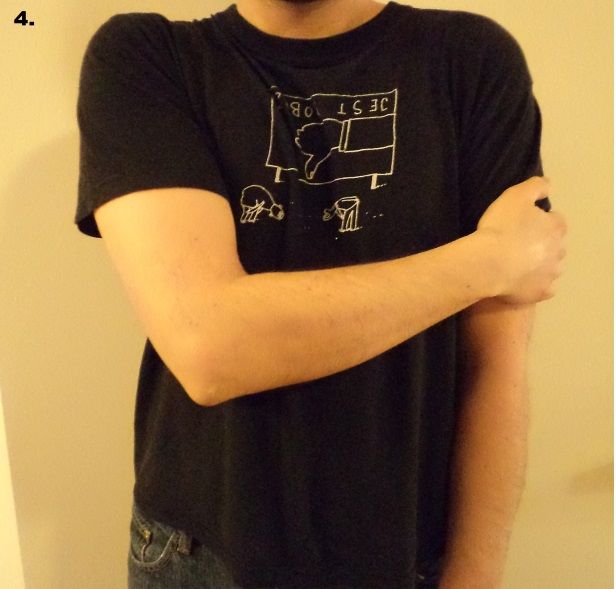 |
| 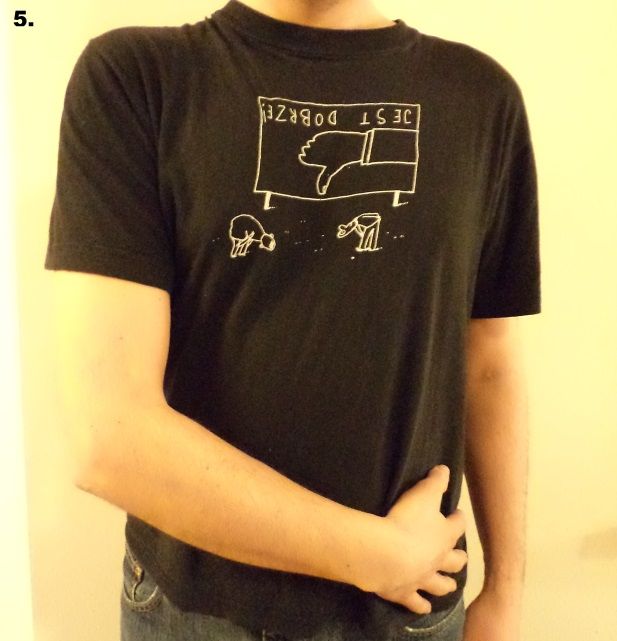 |  |
